# Integrative network analysis for survival-associated gene-gene interactions across multiple genomic profiles in ovarian cancer

**DOI:** 10.1186/s13048-015-0171-1

**Published:** 2015-07-03

**Authors:** Hyun-hwan Jeong, Sangseob Leem, Kyubum Wee, Kyung-Ah Sohn

**Affiliations:** Department of Information and Computer Engineering, Ajou University, Suwon, 443-749 Republic of Korea

**Keywords:** Mutual information, Outcome-associated gene interaction network, Integrative network analysis, Survival analysis, TCGA

## Abstract

**Background:**

Recent advances in high-throughput technology and the emergence of large-scale genomic datasets have enabled detection of genomic features that affect clinical outcomes. Although many previous computational studies have analysed the effect of each single gene or the additive effects of multiple genes on the clinical outcome, less attention has been devoted to the identification of gene-gene interactions of general type that are associated with the clinical outcome. Moreover, the integration of information from multiple molecular profiles adds another challenge to this problem. Recently, network-based approaches have gained huge popularity. However, previous network construction methods have been more concerned with the relationship between features only, rather than the effect of feature interactions on clinical outcome.

**Methods:**

We propose a mutual information-based integrative network analysis framework (MINA) that identifies gene pairs associated with clinical outcome and systematically analyses the resulting networks over multiple genomic profiles. We implement an efficient non-parametric testing scheme that ensures the significance of detected gene interactions. We develop a tool named MINA that automates the proposed analysis scheme of identifying outcome-associated gene interactions and generating various networks from those interacting pairs for downstream analysis.

**Results:**

We demonstrate the proposed framework using real data from ovarian cancer patients in The Cancer Genome Atlas (TCGA). Statistically significant gene pairs associated with survival were identified from multiple genomic profiles, which include many individual genes that have weak or no effect on survival. Moreover, we also show that integrated networks, constructed by merging networks from multiple genomic profiles, demonstrate better topological properties and biological significance than individual networks.

**Conclusions:**

We have developed a simple but powerful analysis tool that is able to detect gene-gene interactions associated with clinical outcome on multiple genomic profiles. By being network-based, our approach provides a better insight into the underlying gene-gene interaction mechanisms that affect the clinical outcome of cancer patients.

## Background

Through the development of high-throughput sequencing technology and collaborative projects such as The Cancer Genome Atlas (TCGA), the integrative analysis of clinical data and genomic data at different molecular levels has emerged as a prominent tool for improving our understanding of the biological mechanisms underlying cancer. Many computational attempts have been made to identify molecular abnormalities that affect clinical outcomes and therapeutic targets, by integrating multiple genomic profiles and clinical data [1–15]. In particular, the association between various genomic features and the clinical outcome of cancer patients has been studied extensively. Previous studies have often focused on the association between each single gene and clinical outcomes [16–19], and have not been able to detect the combined effects of multiple genomic features. Other approaches are based on regression models that can describe the effects of multiple features. For example, the cox regression or sparse regression framework, like elastic net analysis, is effective in finding gene expression signatures associated with the overall survival of cancer patients [20]. However, these methods are limited to detection of the additive effect of multiple features on clinical outcome, and do not translate well for more general types of interaction effects.

More recently, network information either between patients or between genes has been shown to significantly improve the accuracy of predicting clinical outcomes, such as survival in cancer patients. Kim et al., developed an integrated framework by graph-based semi supervised learning, to handle multi-level genomic data for the prediction of clinical outcomes in *ovarian serous cystadenocarcinoma* [10]. The similarity network between patients is first constructed by using genomic feature values, and then the network information is utilized in learning the clinical label of new patients. Cox-regression for predicting cancer patient survival has also been successfully extended to incorporate the network structure among genes [21]. However, many of the existing networks used for such analyses are constructed either by a simple correlation approach between features, or taken from the existing knowledge base, such as protein-protein interaction networks. Neither type of network contains information about the effect of gene interactions on clinical outcomes, from a given dataset. In alternative ways, there were studies to consider effect of clinical outcomes of constructed networks. Vandin et al. proposed mutated sub-networks associated clinical outcome with HotNet algorithm [22, 23], Pauling et al. proposed network integration method with hybrid network construction and differential network mapping for condition specific key pathways [24]. However, these studies focused only interaction or association between single gene and clinical outcomes.

In terms of genomic features, gene signatures based on mRNA expression have been most widely investigated to date, while other features such as Copy-Number Alteration (CNA), miRNA, or methylation levels, are gaining more attention recently. For example, Gorringe et al., tried to identify genomic loci interactions of CNA in samples from ovarian cancer patients, although found no association with survivability [25].

In this paper, we propose a new integrative framework to identify interacting gene pairs that affect the clinical outcome of cancer patients. Our approach of Mutual information-based Integrative Network Analysis (MINA) allows systematic investigation of gene-gene interactions associated with clinical outcome, via gene network construction and analysis. Unlike many existing models, which consider the effects of each single gene or multiple but additive interaction effects on clinical outcome, the proposed method focuses on identifying the gene-gene interaction effect of any type on clinical outcome. By building a gene interaction network, we obtain a global view of the gene interaction landscape that is associated with the clinical outcome of patients. To gain better insight into the gene interactions that affect clinical outcome, we utilized available genomic profiles across different molecular levels. We find that the resulting integrated network has a greatly enhanced level of scale-freeness and biological significance than each network based on a single genomic profile.

Our method is different from many previous computational network analysis schemes in that an edge between genes in our network directly implies the interactive effect of a pair of genes on clinical outcome. For instance, Languino et al. constructed a correlation gene network from data for the NCI-human tumor cell lines [26]. Hong et al. proposed integrative network construction scheme from two independent dataset of ovarian cancer patients [27]. Network-based stratification, which was proposed by Hofree et al. uses mapping scheme from public databases to construct gene-gene interaction network [28]. However, all of those proposed methods constructed networks using information in features only and there was no consideration of the clinical outcome during the network construction. Thus, edges in the networks only represent the strength of interaction between two genes without difference of the outcomes in samples. On the other hand, we proposed an outcome-guided mutual information network in which edges reflect both the interaction effect and difference in the clinical outcome of the given samples. Moreover, the outcome-guided network could improve the survivability prediction performance of the network-based Cox-regression in comparison with traditional networks such as a correlation network or static protein-protein interaction network [29].

Instead of relying on parametric tests, which may suffer from a large number of pairwise tests and multiple testing issues, we use an information-theoretic measure of mutual information and a non-parametric approach to extract significant interactions among genes. Mutual information has been widely used as an association measure in the context of genome-wide association studies for detecting epistasis, but rarely in the association between general genomic features and clinical outcomes. It has the advantages of being flexible and easily applied to both discrete and continuous variables. We implemented an efficient non-parametric testing scheme based on permutation, for measuring the statistical significance of detected interactions.

Here, we apply the proposed method to TCGA data from ovarian cancer patients. Ovarian cancer is a fatal gynecological cancer that is the leading cause of genital system cancer death and fifth-most common fatal cancer among women in the United States [30]. The cancer shows a high recurrence and poor survival rate [31], which cannot be addressed by standard treatment. In this study we detected novel strong pair-wise interactions associated with survival in ovarian cancer, including many genes with little marginal effect. We also present the topological properties and biological significance of networks constructed from multiple genomic profiles.

## Methods

### Mutual information for identifying gene-gene interactions associated with clinical outcome

Using genomic profile data, we identify genomic interactions that are associated with clinical outcome, by utilizing an information-theoretic measure of mutual information [32]. It has been used successfully to detect linear or non-linear association between two random variables [33–36]. In most previous studies for detecting interactions based on mutual information, it has been used as a measure of association between a pair of genes [34, 33]. In other words, focus was on interactions or correlations between genes. We take a different approach by using mutual information to assess the strength of association between a pair of genes and the clinical outcome of given samples. Below, we include a brief description of mutual information and how we modify it to capture genomic interactions associated with clinical outcome.

Entropy of a discrete random variable X is defined as

$$ H(X)=-{\displaystyle {\sum}_{x\in X}p(x){ \log}_2p(x),} $$

and joint entropy of two random variables *X* and *Y* is defined as

$$ H\left(X,Y\right)=-{\displaystyle \sum_{x\in X}}{\displaystyle \sum_{y\in Y}}p\left(x,y\right){ \log}_2p\left(x,y\right). $$

Mutual information of two random variables *X* and *Y* is defined as

$$ I\left(X; Y\right)=H(X)+H(Y)-H\left(X,Y\right). $$

In order to measure the strength of association between a pair of genes and clinical outcome, we use the extended version of mutual information, which is as follows:

$$ I\left({X}_1,{X}_2; Y\right)=H\left({X}_1,{X}_2\right)+H(Y)-H\left({X}_1,{X}_2,,,Y\right). $$

Here, *X*_1_ and *X*_2_ denote random variables for two genes, and *Y*denotes random variables for the clinical outcome of patients.

When a random variable is discrete, its probability distribution can be easily approximated by the frequency of each possible value. If a genomic profile consists of continuous valued features, then it is not straightforward to calculate mutual information directly, because the respective probability distribution for the continuous variable is unknown by given values [37]. To address this, we use the histogram-based technique [34] to discretize continuous values. This technique divides the range of a set of continuous values into equal-sized bins. The binning interval of an *i*-th gene in a genomic profile is determined as $\frac{Max\left({V}_i\right)-Min\left({V}_i\right)}{B}$, where *B* denotes the number of bins and *V*_*i*_ is a continuous-valued vector for the gene in the profile. The size of the vector is the number of samples in the profile. As the result of discretization, a continuous expression value from a profile goes into one of the *B* bins.

We also discretize the clinical outcome variable as binary and divide patients into two groups based on survival months. As in previous studies dealing with binarized clinical information [14, 38], we define the short-term and long-term groups as the patients that survived less than or equal to 36 months, or more than 36 months, respectively.

Discretization of a genomic profile induces a partition on the set of samples. Then entropy of a random variable X can be defined in terms of the partition as follows:

$$ H(X)=-{\displaystyle \sum_{i=1}^n}\frac{\left|{A}_i\right|}{\left|S\right|}{ \log}_2\frac{\left|{A}_i\right|}{\left|S\right|}, $$

where *X* = {*A*_1_, *A*_2_, …, *A*_*n*_} is a partition on the set of samples *S*, i.e. *S* = *A*_1_ ∪ *A*_2_ ∪ ⋯ ∪ *A*_*n*_ and A_i_ ∩ *A*_*j*_ = ∅ for distinct *i* and *j*. Joint entropy of two partitions *X* = {*A*_1_, *A*_2_, …, *A*_*n*_} and *Y* = {*B*_1_, *B*_2_, …, *B*_*m*_} can also be defined as follows:

$$ H\left(X,Y\right)=-{\displaystyle \sum_{i=1}^n}{\displaystyle \sum_{j=1}^m}\frac{\left|{A}_i\cap {B}_j\right|}{\left|S\right|}{ \log}_2\frac{\left|{A}_i\cap {B}_j\right|}{\left|S\right|}. $$

It can be naturally extended to joint entropy of any number of multiple partitions.

### Extraction of outcome-associated gene-gene interactions by permutation test

Since the exact probability distribution of mutual information computed on a dataset is generally unknown, the *p*-value for the significance of a computed mutual information value is not directly available. Instead of using an approximate scheme such as chi-square distribution approximation [39], we use a non-parametric approach based on the permutation strategy in [34] and derive a threshold for the mutual information value. Specifically, clinical outcome labels (short-term vs. long term) are randomly permuted and the mutual information values with respect to the permuted labels are calculated for every pair of genes. We repeat this 30 times and compute the average mutual information across 30 runs by ${I}_{\mathrm{avg}}\left(i,j\right)=\frac{1}{30}{\displaystyle {\sum}_{p=1}^{30}{I}_{\mathrm{avg}}\left({g}_i,\ {g}_j,; {Y}_p\right)}$ for each pair of genes *g*_*i*_ and *g*_*j*_, and *Y*_*p*_ for the permuted clinical outcome labels at *p*-th run.

The threshold θ is determined as the maximum of average mutual information values, i.e., θ = max_*i* ≠ *j*_*I*_avg_(*i*, *j*). The pairs of genes having mutual information above this threshold with respect to the original clinical outcome labels are considered as associated with the clinical outcome and included for further analysis.

### Construction of integrative gene networks

We compute the mutual information for every pair of genes and clinical outcome by using each genomic profile separately and obtain those interactions that are associated with clinical outcome by the proposed method. This results in an outcome-guided mutual information gene network in which two genes are connected if their combination is associated with clinical outcome. We denoted a network for each profile as follows:

$$ {G}_{\alpha}^{profile}=\left\{\left({g}_i,{g}_j\right)\Big|{g}_i,{g}_j\in P\  and\ I\left({g}_i,{g}_j,;,Y\right)\ge \theta \left(1+\alpha \right)\right\} $$

where *g*_*i*_ and *g*_*j*_ are two genes in the set of all genes *P*, *θ* is the threshold from the permutation strategy, and *α* is the parameter for adjusting the statistical significance level. We constructed gene networks by applying the proposed method to each of the mRNA expression, CNA, and methylation profiles, which we denoted as *G*_*α*_^*mRNA*^, *G*_*α*_^*CNA*^ , and *G*_*α*_^*METH*^.

To enhance our view on the gene interaction associated with clinical outcome across multiple genomic profiles, we can further construct an integrated network by merging the three networks. As a pilot study, two types of integrated networks are considered: I^∃^ = *G*^*mRNA*^ ∪ G^*CNA*^ ∪ *G*^*METH*^ (integrated network with one-or-more occurrence of association across profiles) and I^∀^ = *G*^*mRNA*^ ∩ G^*CNA*^ ∩ *G*^*METH*^ (integrated network with co-occurrence of associations in every profile) to figure out the overall characteristic and relation of different genomic profiles*.* Integrated network I^∃^ is a union-set of associations which exists at least in one of the genomic profiles. In contrast, an edge for an association between two genes in I^∀^ must be in every given single profile networks.

### Survival analysis of identified gene pairs

Once we obtain pair-wise gene features associated with the clinical outcome, we perform the following survival analysis to validate the result. For a given pair of genes, the patients are stratified into two groups based on the feature value combination of the selected genes, as in the grouping method of Multifactor-Dimensionality Reduction (MDR) [40, 41]. We first set a threshold *ρ* as the ratio of the number of short-term survival patients to the total number of patients in a given dataset, which was 146/340 in our study. For each possible combination of feature values at the gene pair, we identify patients with the feature combination and examine the ratio of the number of short-term survival patients to the total number of patients among the extracted ones. Each combination of gene feature values is considered as high-risk if the ratio from the combination is above the threshold *ρ*, and otherwise, as low-risk. This stratifies the patients into two groups of high-risk and low-risk, based on the values of gene pairs. We then apply the log-rank test to assess the significance of the difference in survivability by the gene pair. This is performed on the identified gene pairs as well as on each gene for comparison.

### Network analysis

We analyzed the constructed gene networks in terms of the network topologies and then in terms of the biological functionality through functional enrichment test. As many previous studies have revealed the scale-freeness of gene networks [42, 2, 43–46], we examined the scale-freeness of the constructed gene networks along with other topological properties at each significance level. In a scale-free network, the distribution *p*(*k*) of the node degrees follows a power law *p*(*k*) ~ *k*^− *γ*^, where *p*(*k*) is the frequency of the node whose degree is *k*. To measure scale-freeness of a network, Zhang and Horvath [45] proposed to use the coefficient of determination *R*^2^, which is the model-fitting index of the linear model that regresses log *p*(*k*) on log *k*. If *R*^2^ is close to 1.0, the network is considered scale-free. For a network constructed from each genomic profile and for each significance level with varying parameter values of α = 0.0, 0.1, 0.5, 0.8, and 1.0, we measured the number of nodes, the number of edges, the number of connected components, the size of the largest component, and the measure of scale-freeness *R*^2^.

We performed enrichment analysis on the obtained networks to assess common or related biological functionalities of the genes belonging to the same connected component of the network. We ran gene ontology (GO) [47] enrichment analysis for the network in Cytoscape [48] with Biological Network Gene Ontology tool (BINGO) [49]. We used Ontology and annotation data in (http://www.geneontology.org/). We ran those analysis for the co-occurrence network, the one-or-more occurrence network, and each of the three networks constructed by using each profile separately.

### MINA: mutual information based network analysis framework

**Fig. 1:**
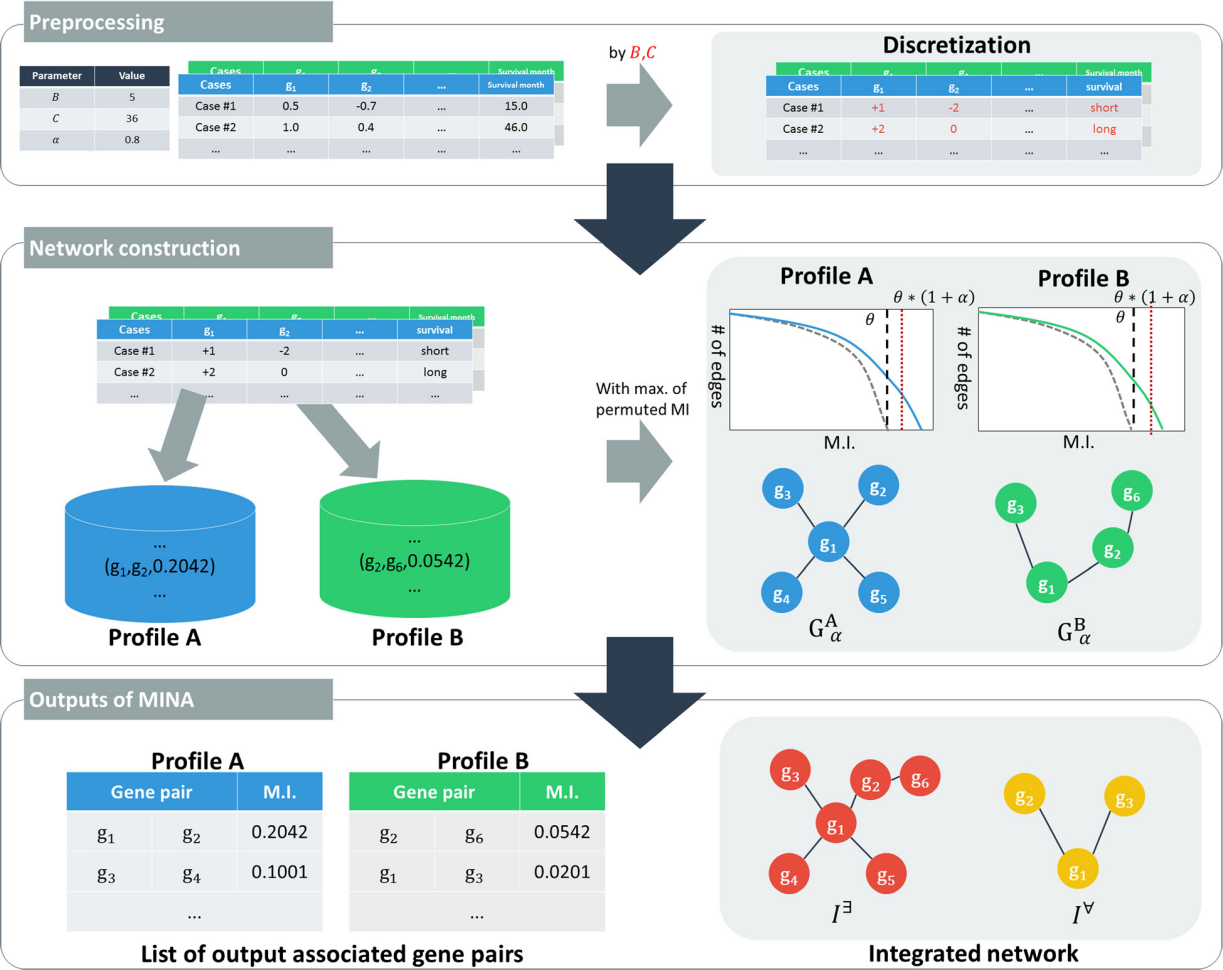
Illustration of MINA

We developed a tool named MINA that automates the process of identifying significant gene interactions associated with clinical outcome and of generating various networks from those pairs. Figure 1 illustrates the overall process performed inside MINA. Genomic profiles, clinical outcomes, and the model parameters (B, C, and α) are used as the input. MINA then transforms continuous feature values that may exist in some genomic profiles (e.g., mRNA expression or methylation) and clinical outcome to discrete value based on the parameters B (the number of bins) and C (threshold for survival months) and calculate mutual information value for every possible pair of genes. This tool then outputs significant pairs of genes for a given genomic profile and the resulting networks.

MINA is written in C++ and runs on operating system based on UNIX. We also used OpenMP (Open Multi-Processing) (http://www.openmp.org), a parallel processing library, to hasten the overall process. For the TCGA dataset, it took about 2 to 3 h to run the entire process in a common desktop computer. The source codes for MINA are publically available at https://github.com/hhjeong/MINA.

## Results

### Ethics statements

All data related to human subjects used for this study is de-identified and publicly available from The Cancer Genome Atlas project (http://cancergenome.nih.gov/). Therefore, this research is not classified as a human subject research and no Institutional Review Board approval is required.

### TCGA data and pre-processing

We used genomic and clinical profiles of patients with ovarian serous cystadenocarcinoma from TCGA to demonstrate our proposed method. The genomic profiles included mRNA expression (mRNA), copy number alteration (CNA), and methylation (METH). We initially focused on the genomic features of 20,642 genes in the protein-coding region of 575 patients. The clinical information for the patients was also extracted. All datasets were downloaded from cBioPortal [50, 51] (http://www.cbioportal.org) that provides convenient data acquisition tools for TCGA data. Table 1 summarizes platforms and data types used in our study. We further pre-processed the datasets to filter out genes or patients and to discretize the data as described below.

**Table 1:** Summary of datasets used in this study

Genomic profile	Platform	Data type
mRNA	Agilent microarray	Continuous
CNA	Affymetrix SNP 6	Discrete
methylation	Illumina Infinium HumanMethylation27	Continuous

We applied a two-step procedure to filter genes and patients. In the first step, the following three filters were applied sequentially. First, each gene with missing values across the patient group was removed from all genomic profiles. Then, each patient with all missing values for the remaining genes was removed from all profiles. Finally, each gene with a missing value in at least one of the three profiles on the remaining patients was removed. Thus, we had 10,022 protein-coding genes in common across the three profiles of mRNA expression, DNA methylation, and copy number alteration.

As our analysis employed clinical information as a binary outcome of short-term versus long-term survival, in the second filtering step, we further excluded patients whose label assignments were ambiguous from the analysis. That is, the patients with no survival status or with a survival status as *living* and observed survival time of <36 months were filtered out in the second step. As a result, we had 146 patients in the short-term group and 194 patients in the long-term group.

The copy number alteration profile had discrete valued features with five values of −2, −1, 0, 1, and 2, and therefore, we directly used this representation from GISTIC [52] to compute mutual information. We discretize mRNA expression and DNA methylation profiles as described before with the parameter for the number of bins *B* = 5 to be consistent with CNA profile.

### Distribution of mutual information on each genomic profile

**Fig. 2:**
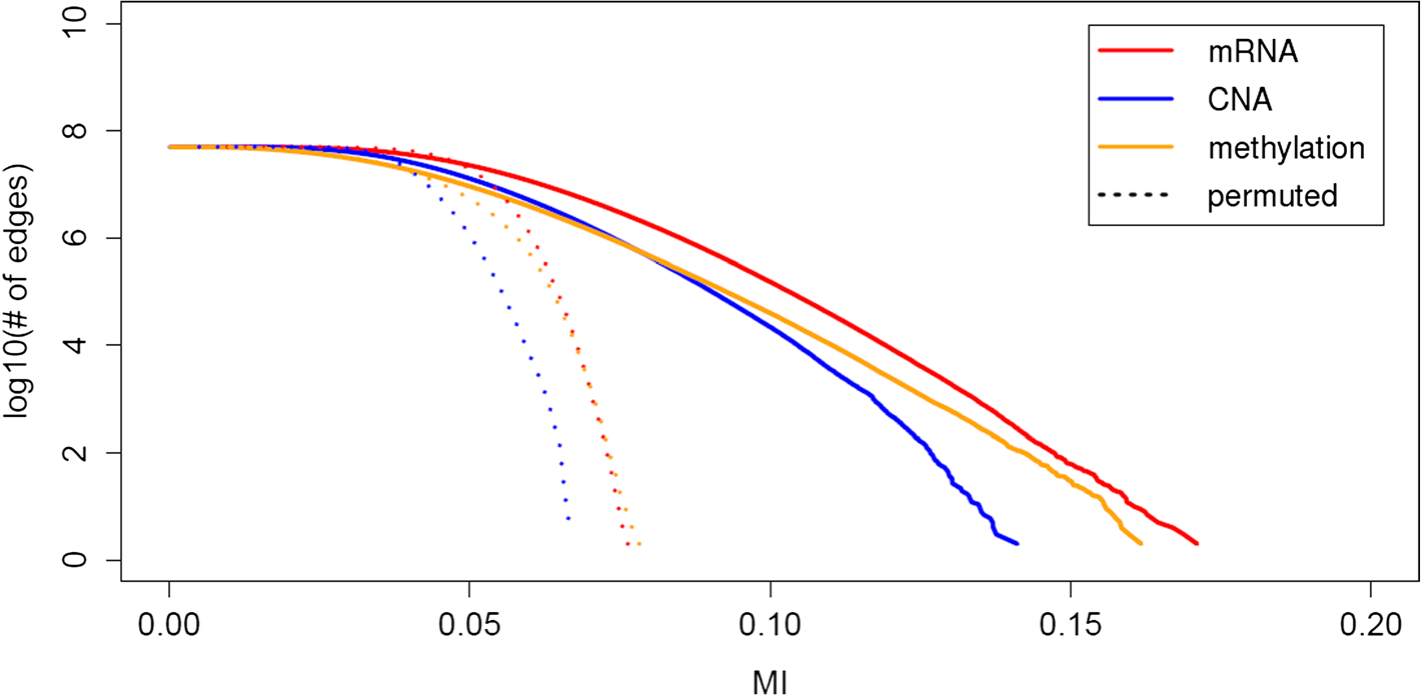
Empirical distribution of mutual information values. We show the distribution of mutual information values computed for every pair of genes in each profile of mRNA expression (*red*), CNA (*blue*) and methylation (*yellow*). The *solid lines* correspond to the values with respect to the original clinical outcome labels, and the *dotted lines* are with respect to the permuted labels averaged over 30 permutations

We calculated mutual information values using the original and permuted clinical outcome labels of patients, for every pair of genes on each genomic profile in TCGA datasets. Figure 2 shows the empirical distribution of mutual information computed on each real profile (mRNA, CNA, METH) used in this study. The solid lines are with respect to the original clinical outcome labels, and the dotted lines are with respect to the permuted labels averaged over 30 runs. The results from the permuted labels could not create mutual information above 0.0763, 0.0664, and 0.0782 on mRNA, CNA, and methylation profiles, respectively. Therefore, we set these numbers as threshold mutual information θ for each profile separately. A pair of genes with mutual information above this threshold was considered to be associated with clinical outcome.

Gene interactions associated with clinical outcome occur more typically with respect to mRNA expression or copy number alteration levels, but less so with respect to methylation levels. The mRNA expression profile produced the highest number of gene pairs (2,562,178). The CNA profile was second with 2,472,048 pairs, and the methylation profile had far fewer interactions with 554,048 gene pairs (Table 2). This corresponds to about 1–5 % of all pairs of genes (i.e., out of 5 × 10^7^ pairs). When we increase the significance level by setting the threshold as θ × (1 + α) and varying α = 0.0, 0.1, 0.5, 0.8 and 1.0, the number of remaining edges (or gene pairs) becomes substantially less. For example, when α = 0.5, the numbers of gene pairs are 20,219, 23,143, and 3,641, for mRNA expression, CNA, and methylation profiles, respectively. The overall result is summarized in Table 2.

**Table 2:** Threshold mutual information on each genomic profile

Genomic profile	Threshold		No. of gene pairs above threshold	Percentage
	α	θ (1 + α)		
mRNA	0.0	0.0763	2,562,178	5.10 %
	0.1	0.0839	1,125,398	2.24 %
	0.5	0.1145	20,219	0.04 %
	0.8	0.1373	555	<0.001 %
	1.0	0.1526	45	<0.001 %
CNA	0.0	0.0664	2,472,048	4.92 %
	0.1	0.0730	1,090,500	2.17 %
	0.5	0.0996	23,143	0.05 %
	0.8	0.1195	526	<0.001 %
	1.0	0.1328	17	<0.001 %
METH	0.0	0.0782	554,048	1.10 %
	0.1	0.0860	221,680	0.44 %
	0.5	0.1173	3,641	0.01 %
	0.8	0.1407	115	<0.001 %
	1.0	0.1564	8	<0.001 %
Total			50,215,231	100.00 %

### Survival analysis of selected pair-wise genes

**Fig. 3:**
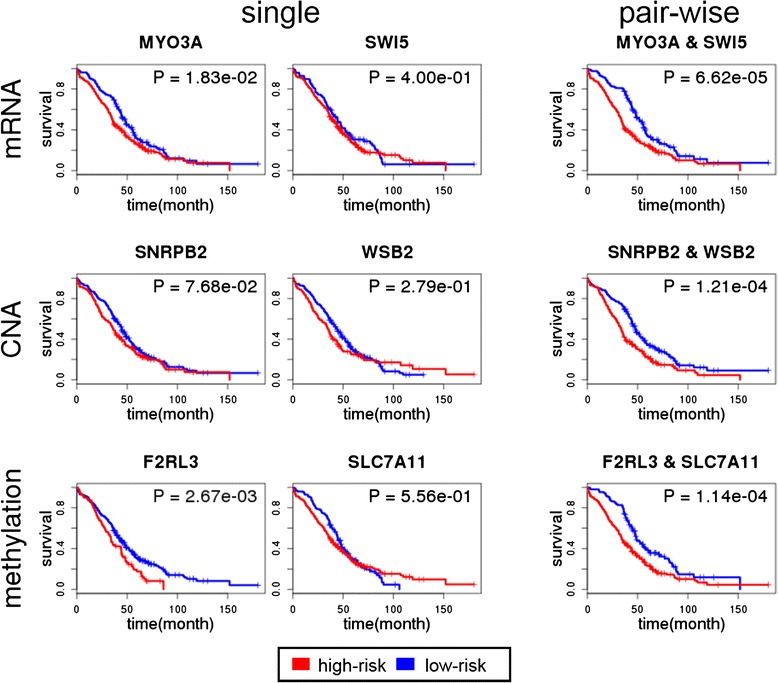
Kaplan-Meier survival plots of the gene pair with the highest mutual information value for each single profile. We show the Kaplan-Meier survival curve of the gene pair having the highest mutual information along with the ones derived by each single gene

We validated the significance of identified gene interaction effects on clinical outcome by applying the survival analysis described in Methods. Table 3 shows the results of the log-rank test applied to the top 10 gene pairs from each genomic profile. All of the top 10 gene pairs induced a significant difference in survival, with *p*-values ranging from 1.67 × 10^− 3^ to 5.08 × 10^− 7^ across different profiles. In Fig. 3, the Kaplan-Meier survival curve of the gene pair that has the highest mutual information is shown for each profile, along with the ones derived by each single gene. The top pair of genes from the mRNA expression profile was MYO3A, a previously identified cancer gene [53] and SWI5, a recombination repair homolog. The *p*-value from the log-rank test for survival difference according to the gene pair was 6.62 × 10^− 5^, while each single gene produced *p*-values of 0.02 (MYO3A) and 0.4 (SWI5). In the case of the CNA profile, the top pair was from SNRPB2 and WSB2, both cancer genes documented in COSMIC [54], with a *p*-value of 1 .21 × 10^− 4^, whereas the *p*-value based on each gene separately was 0.08 and 0.3, respectively.

**Table 3:** Top 10 gene pairs for each genomic profile

Genomic profile	Gene pair		Chromosome		Mutual information	*p*-value
mRNA	MYO3A	SWI5	10p11.1	9q34.13	0.1753	6.62E-05
	CYTH3	ZC3H14	7p22.1	14q31.3	0.1710	8.70E-08
	ARHGDIA	DNMBP	17q25.3	10q24.31	0.1688	1.81E-05
	AK1	THBS1	9q34.1	15q15	0.1670	3.82E-07
	MCM3	PCDHB5	6p12	5q31	0.1645	1.20E-05
	CRYAB	TTPAL	11q22.3-q23.1	20q13.12	0.1627	1.57E-07
	CYP39A1	NUAK1	6p21.1-p11.2	12q23.3	0.1627	2.01E-08
	CMBL	KRT23	5p15.2	17q21.2	0.1624	1.67E-03
	CYTH3	FBXW8	7p22.1	12q24.23	0.1616	4.66E-06
	CYTH3	IDE	7p22.1	10q23-q25	0.1605	4.16E-08
CNA	SNRPB2	WSB2	20p12.1	12q24.23	0.1432	1.21E-04
	KIF16B	WSB2	20p11.23	12q24.23	0.1411	1.52E-04
	SNRPB2	TAOK3	20p12.1	12q	0.1377	1.70E-04
	SNRPB2	TESC	20p12.1	12q24.22	0.1372	1.22E-04
	PEBP1	SNRPB2	12q24	20p12.1	0.1370	1.70E-04
	NOS1	SNRPB2	12q24.22	20p12.1	0.1367	1.22E-04
	KIF16B	TAOK3	20p11.23	12q	0.1355	2.13E-04
	KIF16B	TESC	20p11.23	12q24.22	0.1352	1.53E-04
	KIF16B	PEBP1	20p11.23	12q24	0.1349	2.13E-04
	FBXW8	SNRPB2	12q24.23	20p12.1	0.1348	1.87E-04
METH	F2RL3	SLC7A11	19p12	4q28-q32	0.1670	1.14E-04
	CCM2L	TMEM129	20q11.21	4p16.3	0.1618	2.60E-04
	CAND1	YTHDC1	12q14	4q13.3	0.1598	5.86E-04
	ENSA	PTHLH	1q21.3	12p12.1-p11.2	0.1584	2.59E-06
	CDH8	DYRK2	16q22.1	12q15	0.1582	5.08E-11
	FOXL1	NRTN	16q24	19p13.3	0.1575	1.55E-07
	FOLR2	TMEM129	11q13.3-q14.1	4p16.3	0.1570	1.04E-05
	SYT8	ZBTB1	11p15.5	14q23.3	0.1566	1.91E-04
	IL23A	ZBTB1	12q13.13	14q23.3	0.1559	3.62E-06
	MFAP4	ZBTB1	17p11.2	14q23.3	0.1557	3.51E-05

**Fig. 4:**
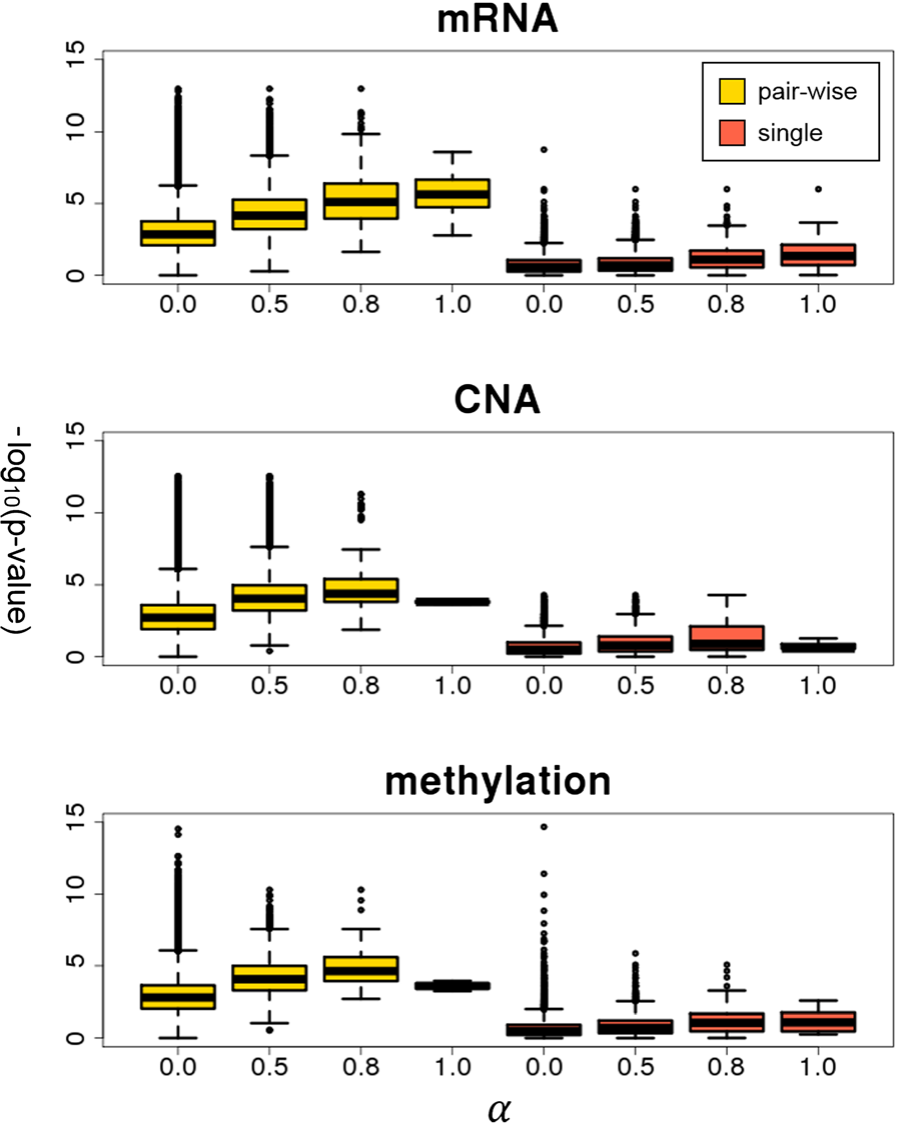
Boxplots for *p*-values from survival analysis. The distribution of *p*-values from the survival analysis for the extracted gene pairs obtained from different significance levels of α is shown as a boxplot

For more comprehensive analysis, we ran the survival analysis for all the extracted gene pairs obtained from four different significance levels of α = 0.0, 0.5, 0.8 and 1.0. The distribution of the resulting *p*-value is shown in Fig. 4 as a box plot. For comparison, we also included the box plots for *p*-values for each single gene in the identified gene pairs. Overall, the association significance was substantially stronger in the case of gene pairs than in single genes, across different profiles and parameter settings. This means that there are many genes having weak or no effects, but a strong interaction effect on clinical outcome. Moreover, at each parameter α, the most significant *p*-value becomes much larger, that is, −log(*p*-value) becomes much smaller when we consider the single genes separately, in the case of mRNA and CNA profiles. The methylation profile behaved differently in that the top *p*-value at α = 0.0 was very similar in both the pairwise and single analyses. It appears that the gene-gene interaction at the methylation level is not as prominent as in other profiles, and the top interaction effects are largely based on the marginal effects of single genes.

### Outcome-guided mutual information gene networks

We constructed outcome-guided mutual information gene networks by considering genes as nodes, and connecting two gene nodes if their combination was significantly associated with clinical outcome. For a network constructed from each genomic profile and also for each significance level with varying parameter values of α = 0.0, 0.1, 0.5, 0.8, and 1.0, we measured the number of nodes, the number of edges, the number of connected components, the size of the largest component, and the measure of scale-freeness *R*^2^ (Table 4).

**Table 4:** Network Topologies for different α values

α	Profile	Vertices	Edges	Number of components	Size of largest component	*R* ^*2*^
0.0	mRNA	9,997	2,562,178	1	9,997	0.643
	CNA	10,021	2,472,048	1	10,021	0.590
	METH	9,801	554,048	1	9,801	0.839
	I^∀^	1,244	1,538	61	1,105	0.914
	I^∃^	10,022	5,385,486	1	10,022	0.366
0.1	mRNA	9,943	1,125,398	1	9,943	0.758
	CNA	9,934	1,090,500	1	9,934	0.749
	METH	9,118	221,680	1	9,118	0.842
	I^∀^	138	95	44	27	0.950
	I^∃^	10,022	2,396,372	1	10,022	0.505
0.5	mRNA	6,466	20,219	25	6,418	0.810
	CNA	2,886	23,143	9	2,855	0.831
	METH	2,166	3,641	25	2,116	0.700
	I^∃^	8,032	46,975	11	8,012	0.864
0.8	mRNA	641	555	112	358	0.804
	CNA	245	526	10	106	0.892
	METH	145	115	32	58	0.690
	I^∃^	1,002	1,196	137	579	0.913
1.0	mRNA	73	45	28	11	0.797
	CNA	13	17	1	13	0.363
	METH	15	8	7	3	1.000
	I^∃^	100	70	35	23	0.803

Overall, networks based on mRNA expression and CNA profiles tended to have a larger value of *R*^2^ as α increases, with the maximum at α = 0.8. The networks based on the methylation profile tended to have smaller *R*^2^ when we increased α. We then examined the I^∀^ and I^∃^ at each setting. The number of gene interactions appearing across all three profiles was relatively small. For example, at α = 0.1, the number of edges in I^∀^ was only 95, while the one-or-more occurrence network (I^∃^) at the same significance level had more than 2 million edges. There was no common edge across all of the profiles at a significance level of 0.5 or higher. Also, we did not find a shared edge between any pair of profiles at a significance level 0.8 or higher.

**Fig. 5:**
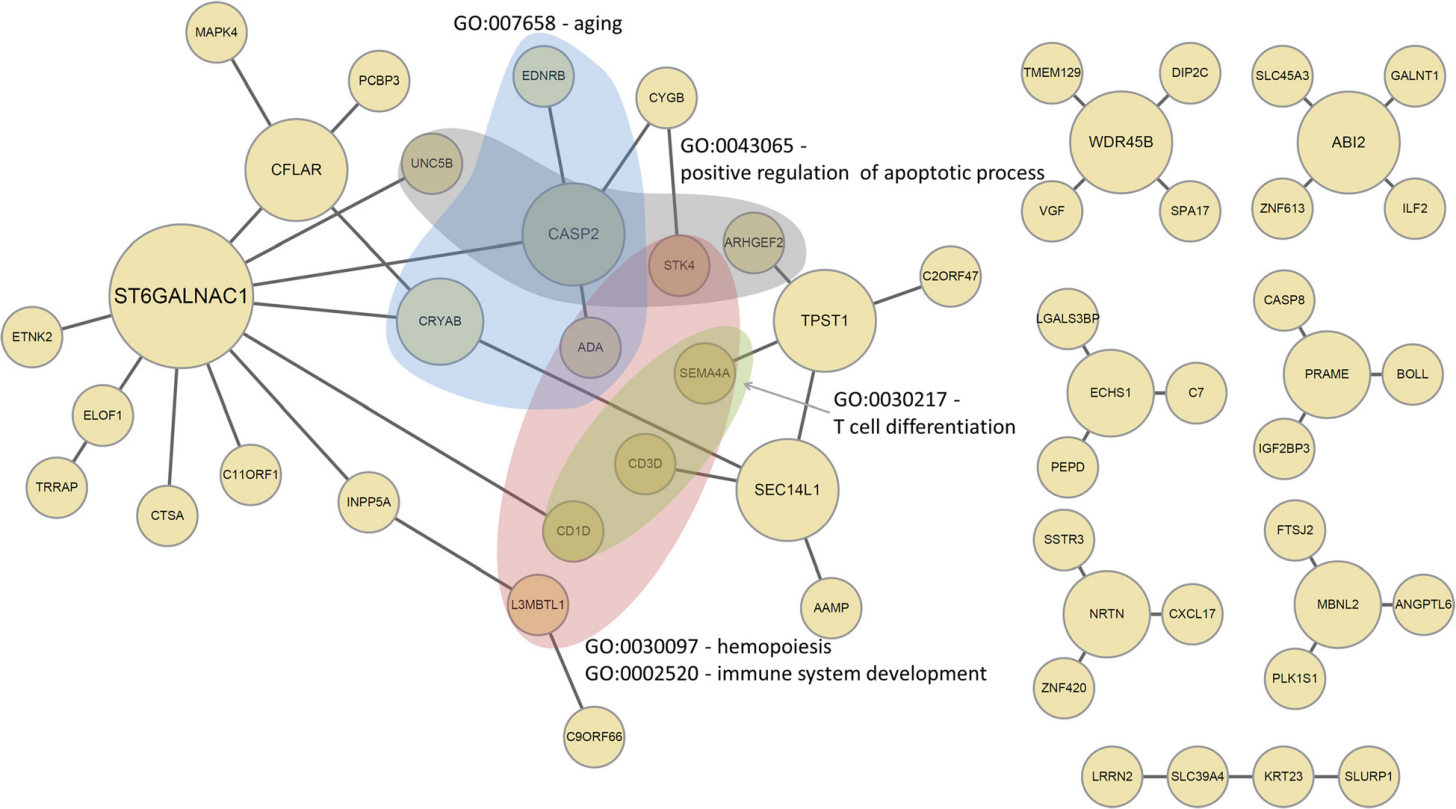
I_0.8_^∀^ of whole genomic profiles

**Fig. 6:**
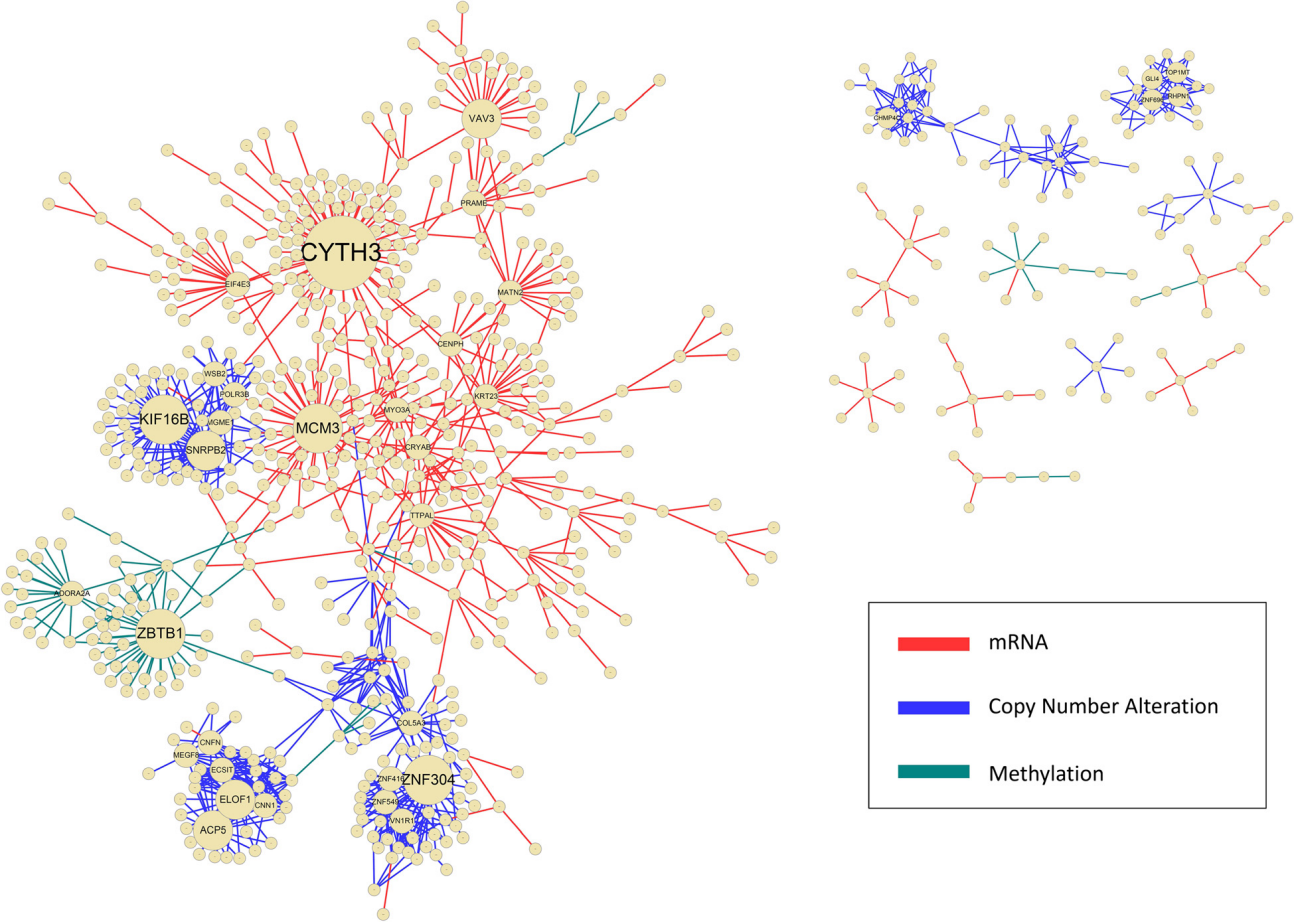
I_0.8_^∃^ of whole genomic profiles

Interestingly, the integrated network, either by taking the intersection or the union of edges, appeared to have a significantly enhanced scale-freeness. The co-occurrence network I_0.1_^∀^ had the highest *R*^2^ value of 0.950, and the one-or-more occurrence network with I_0.8_^∃^ had the second highest *R*^2^ value of 0.913. This may suggest that integrated networks are more effective in identifying functional gene modules across multiple molecular levels than networks constructed by using each profile separately. We selected these two networks to run further analysis. The graphical representation of the selected intersection network and the union network is shown in Fig. 5 and Fig. 6, respectively.

We performed gene ontology (GO) enrichment analysis to assess common or related biological functions of the genes belonging to the same connected component of the constructed network. We ran the analysis for each of the three networks based on mRNA, CNA, and methylation profiles, and for their one-or-more occurrence network at α = 0.8. The co-occurrence network at α = 0.1 was analyzed due to its superior scale-freeness and network sparseness at a higher significance level.

**Fig. 7:**
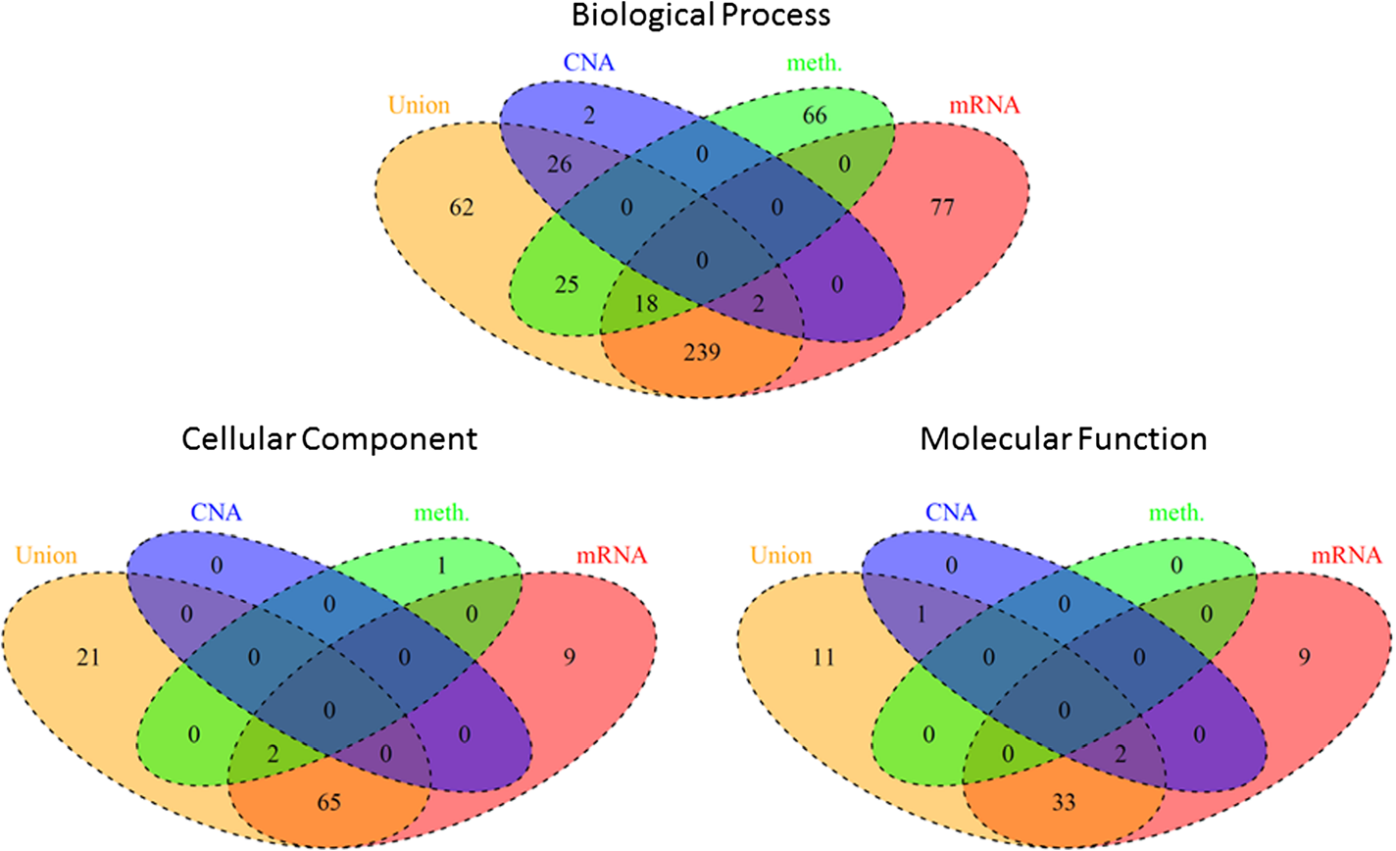
Four-way Venn diagram summarizing the number of shared and unique GO terms enriched in the network from each profile

We first compared the number of enriched GO terms from each constructed network (Fig. 7). The mRNA profile revealed the greatest number of significant terms among the single networks, which was expected. There was no shared GO term between the CNA and methylation profiles, which may suggest distinct functional roles for each profile on clinical outcome. I_0.8_^∃^ indicated the greatest number of enriched GO terms with 62 additional BP (Biological Process), 21 CC (Cellular Component), and 11 MF (Molecular Function) terms, which were not found in networks constructed by any of the single genomic profiles. Therefore, the integration of networks may provide a better insight into the gene interaction landscape associated with clinical outcome.

We further investigated the genes in the largest component of I_0.8_^∃^, which were enriched with 176 GO terms (112 BP, 42 CC, and 22 MF terms). The five most significant GO terms in the largest component were poly(A) RNA binding (GO:0044822), nucleoplasm (GO:0005654), extracellular vesicular exosome (GO:0070062), apoptotic process (GO:0006915), and protein ubiquitination (GO:0016567). These GO terms are closely related to ovarian cancer, based on previous studies. For example, apoptotic process is a cell death term, and Jäättelä reported that defects in apoptotic signaling pathways are common in cancer cells [55]. In addition, protein ubiquitination is a highly relevant term as ubiquitin-mediated proteins have an important role in the mutation of a target oncogene [56]. Table 5 summarizes significantly enriched GO terms with the corresponding *p*-values for the largest connected component of the I_0.8_^∃^. To present more specific functionality, we show the term at the lowest level from the root of the directed acyclic graph for each GO category if multiple terms along the same path from the root are found to be significant.

**Table 5:** Significantly enriched GO terms in the largest component of I_0.8_^∃^

Category	ID	Description	*p*-value	Adjusted *p*-value (FDR)	Count	Total
MF	GO:0044822	poly(A) RNA binding	4.37E-12	1.35E-09	50	1180
CC	GO:0005654	nucleoplasm	1.93E-09	6.54E-08	63	1745
CC	GO:0070062	extracellular vesicular exosome	2.39E-09	7.09E-08	59	1589
BP	GO:0006915	apoptotic process	1.32E-07	1.20E-05	49	1305
BP	GO:0016567	protein ubiquitination	1.75E-07	1.52E-05	28	542
CC	GO:0005730	nucleolus	5.61E-06	1.06E-04	38	1072
CC	GO:0031226	intrinsic component of plasma membrane	1.66E-05	2.89E-04	49	1612
BP	GO:0006366	transcription from RNA polymerase II promoter	1.60E-05	6.57E-04	26	611
CC	GO:0005887	integral component of plasma membrane	5.07E-05	8.34E-04	46	1546
BP	GO:0071156	regulation of cell cycle arrest	2.62E-05	9.70E-04	10	116
BP	GO:0001775	cell activation	5.63E-05	1.86E-03	31	856
BP	GO:0045087	innate immune response	7.46E-05	2.34E-03	34	993
CC	GO:0000228	nuclear chromosome	1.60E-04	2.43E-03	19	453
MF	GO:0042803	protein homodimerization activity	4.87E-05	3.26E-03	26	781
MF	GO:0019901	protein kinase binding	4.93E-05	3.26E-03	22	603
MF	GO:0008201	heparin binding	5.51E-05	3.40E-03	11	182
BP	GO:0071901	negative regulation of protein serine/threonine kinase activity	1.29E-04	3.69E-03	10	140
BP	GO:0007596	blood coagulation	1.36E-04	3.80E-03	22	541
CC	GO:0005783	endoplasmic reticulum	3.16E-04	4.27E-03	51	1918
BP	GO:0051222	positive regulation of protein transport	1.88E-04	4.88E-03	15	301
BP	GO:0000086	G2/M transition of mitotic cell cycle	2.27E-04	5.70E-03	10	150

We also found that major hub genes of the I_0.8_^∃^ network are related with ovarian cancer-related pathways. For example, *Cytohesin 3* (CYTH3), the first hub having the largest number of neighbors in the network, is involved in the PI3K pathway (M14532) in MSigDB [57]. This pathway is a common drug target of human cancer, including ovarian cancer [58, 59]. Furthermore, *Minichromosome maintenance complex component 3* (MCM3), the third hub, is included in the cell cycle pathway (hsa04110) [60], which is important to the cancer research because alterations in the mechanism characterize the abnormal proliferation of human malignant tumors [61]. Previous research also reported that the cell cycle arrest in the G2/M phase via the blockade of cyclin B1/CDC2 in human ovarian cancer cells [62]. From this observation, we presume that interactions of major hub genes with connected neighbors can play an important role in determining the overall survival of ovarian cancer patients.

For the I^∀^, many BP terms were discovered in the largest connected-component, but not from CC or MF categories. Table 6 shows the most significant GO terms for the largest connected-component of the co-occurrence network. The 5 most significant GO terms were hemopoiesis (GO:0030097), immune system development (GO:0002520), aging (GO:0007568), T cell differentiation (GO:0030217) and positive regulation of apoptotic process (GO:0043065). Immune system development and T cell differentiation are terms corresponding to the immune system, which has a significant role in cancer development and progression [63]. Positive regulation of apoptotic process is a cell death term, and is enriched in genes regulated by Ubiquitin carboxyl terminal hydrolase 1 (UCHL1) [64], which is a putative tumor suppressor in ovarian cancer. The hub genes also have known roles in cancer progression. For example, the top hub gene in the network was ST6GALNAC1 which is known to have an important role in ovarian cancer [65].

**Table 6:** Significantly enriched GO terms in the largest component of I_0.1_^∀^

Category	ID	Description	*p*-value	Adjusted *p*-value (FDR)	Count	Total
BP	GO:0030097	hemopoiesis	1.82E-05	6.81E-03	6	699
BP	GO:0002520	immune system development	4.12E-05	6.81E-03	6	809
BP	GO:0007568	aging	3.03E-04	1.36E-02	4	399
BP	GO:0030217	T cell differentiation	4.69E-04	1.99E-02	3	185
BP	GO:0043065	positive regulation of apoptotic process	7.47E-04	2.02E-02	4	507
BP	GO:0006915	apoptotic process	5.92E-04	2.02E-02	6	1320
BP	GO:0001890	placenta development	1.07E-03	2.44E-02	3	246
BP	GO:0050870	positive regulation of T cell activation	1.08E-03	2.44E-02	3	247
BP	GO:0023014	signal transduction by phosphorylation	1.49E-03	2.90E-02	3	276
BP	GO:0071214	cellular response to abiotic stimulus	1.68E-03	2.93E-02	3	288
BP	GO:0001525	angiogenesis	4.53E-03	4.90E-02	3	409

## Discussions

We have proposed a new network-based analysis framework to detect gene pairs associated with the clinical outcome and to analyze the resulting networks systematically. Our survival analysis showed that there are a large number of gene pairs that are significantly associated with survival in ovarian cancer in which each single gene has very weak or no association. From the integration of the profiles, we also showed that networks constructed by combining information across different genomic profiles had better scale-freeness and revealed more biological significance than a network that was constructed by using only one genomic profile.

In our analysis, the co-occurrence network consisted of a moderate level of interactions in single genomic profiles, but integration of the interactions revealed high biological significance in terms of GO BP terms. In contrast to the I_0.1_^∀^, the I_0.8_^∃^ consisted of stronger interactions for each genomic profile, and significant CC and MF terms were enriched. Interestingly, networks from interactions with high association strength at each profile did not have any shared edges. We also found that sub-networks in the I_0.8_^∃^, which were connected by interactions of mRNA and methylation, had many hubs connected to many peripheral nodes, but sub-networks from CNA had a tendency to interconnect genes without any dominant hub gene structure.

In this study, we took a simple network integration scheme, which showed enhanced network properties despite its simplicity. A more complicated network integration scheme may be employed in our future analyses, such as that used in similarity network fusion using multiple genomic datasets [15]. Besides, we plan to investigate the detection power and robustness of the proposed method through extensive simulation study and real data experiments. Another extension includes the application of the integrative network to network-based Cox-regression method using heterogeneous types of data. We expect that this application would enhance the prediction power and help to understand the complex interaction between different types of genomic profiles for the survivability of cancer patients.

## Conclusions

In this paper, we have proposed a simple but powerful method to detect gene pairs that are associated with the clinical outcome. By being network-based, our approach could provide a better insight into the underlying gene-gene interaction mechanisms that affect the clinical outcome of cancer patients.
